# Prometheus’ fire and Brazilian universities: A decade of the quota law

**DOI:** 10.1016/j.clinsp.2026.101058

**Published:** 2026-07-28

**Authors:** Lucas Leite Cunha, Laura Sterian Ward

**Affiliations:** a Emergency Medicine and Evidence-based Medicine Unit, Escola Paulista de Medicina, Universidade Federal de São Paulo, São Paulo, SP, Brazil; b A. C. Camargo Cancer Center, São Paulo, SP, Brazil; c Laboratory of Cancer Molecular Genetics, Faculdade de Ciências Médicas, Universidade de Campinas, Campinas, SP, Brazil

In August 2012, Brazil enacted Law n° 12,711/2012, establishing affirmative action policies ‒ social, racial, and disability-based quotas ‒ for admission to federal universities. At the time, we (the same authors) published an editorial in this journal expressing concern that the law would undermine university autonomy by removing from institutions the prerogative to define their own admission criteria.^1^ We then turned to Homer's Odyssey as our guiding metaphor. Today, more than a decade later, Prometheus offers a more accurate reflection of what has transpired.

According to Greek mythology, Zeus believed that granting humanity fire and knowledge would result in chaos. Prometheus defied him, stole the flame, and returned it to humankind, for which he was eternally punished. The gods were wrong. Through fire, humanity has discovered art, medicine, politics, and democracy. Without it, civilization would not exist. The quota law, like Prometheus' gift, has proven more illuminating than catastrophic, but we must also reckon honestly with the burdens it has brought. This reckoning requires a distinction we did not make in 2012.

Legally, the law constrained university autonomy ‒ federal institutions lost the right to define their own admission criteria, which were replaced by a uniform national mandate. However, the feared collapse did not occur functionally. Universities adapted. Brazilian institutions proved to be more resilient than we anticipated. The feared collapse of academic standards did not occur. Both statements are true, and intellectual honesty requires that they be held together.

By 2022, Brazil's Higher Education Census had recorded 55,371 students entering federal institutions based on ethnic-racial criteria.^2^ Before 2010, Black and Brown students combined represented 10.7% of higher education enrollment against 19.8% for White students; by 2019, that figure had risen to 38.2%, approaching the White student proportion of 42.5%.^3^ The magnitude of this shift is striking. However, because the same period saw the REUNI program substantially expand federal university capacity, this descriptive trend cannot be attributed to the Quota Law alone. While we cannot isolate the law's unique contribution, the scale of change suggests that it played a significant role alongside REUNI.

At this point, the evidence is clear: the feared academic collapse did not occur. Across a growing body of institutional studies, quota students perform comparably to their non-quota peers in many programs. Family income ‒ not admission category ‒ emerges as the primary predictor of academic outcomes.^4^, ^5^, ^6^ Performance gaps persist in highly competitive engineering and exact-science programs, where entrance-score differentials are largest.^4^ However, these gaps are the legacy of pre-university inequality, not the quota mechanism itself.

However, the fire of Prometheus did not come without a cost, and that cost was not unforeseen. The psychosocial burden on quota students ‒ the daily navigation of stigma, constant questioning of intellectual legitimacy, and struggle to belong in spaces not designed for them^7^ ‒ was widely debated at the law's passage, not discovered later. Financial hardship further compounds this stigma.^8^ Aggregate dropout rates in some institutions favor quota students, but persistence comes at a high, hidden psychosocial cost that raw graduation numbers cannot capture. Likewise, the persistence of structural disadvantage in elite mathematics-intensive programs is not a mysterious side effect of policy. It is the direct expression of inequalities in basic education that the Quota Law was never designed to remedy alone. These are not the contents of Pandora's box opened by surprise; they were predictable from the beginning. These are the structural challenges of implementing access policies in a profoundly unequal society, and they demand a targeted institutional response: academic support, psychological services, and sustained financial assistance ‒ not merely the guarantee of a seat.

The ultimate reach of the fire remains unknown. We know very little about what happens after graduation. Do quota students access postgraduate education at comparable rates to non-quota students? Do they reshape Brazil's professional and academic elite and interrupt the cycles of intergenerational poverty? The law's legacy cannot be fully assessed without longitudinal cohort data following students into the labor market. Generating this evidence is an urgent priority.

In 2012, we feared Prometheus. In 2026, we recognize his gift, though not without reservation. The fire he brought required human ingenuity to sustain and channel productively; left untended, it consumes rather than illuminates the world. Tended well, it illuminates. A decade after the quota law, Brazil has chosen to tend to the fire. This milestone has been reached, but the journey is not yet over. The task now is to ensure that the flame burns evenly for all those who gather around it.

## Data availability

The datasets generated and/or analyzed during the current study are available from the corresponding author upon reasonable request.

## Declaration of competing interest

The authors declare no conflicts of interest.
